# Azidobupramine, an Antidepressant-Derived Bifunctional Neurotransmitter Transporter Ligand Allowing Covalent Labeling and Attachment of Fluorophores

**DOI:** 10.1371/journal.pone.0148608

**Published:** 2016-02-10

**Authors:** Thomas Kirmeier, Ranganath Gopalakrishnan, Vanessa Gormanns, Anna M. Werner, Serena Cuboni, Georg C. Rudolf, Georg Höfner, Klaus T. Wanner, Stephan A. Sieber, Ulrike Schmidt, Florian Holsboer, Theo Rein, Felix Hausch

**Affiliations:** 1 Max Planck Institute of Psychiatry, Clinical Department, Munich, Germany; 2 Max Planck Institute of Psychiatry, Department of Translational Research in Psychiatry, Munich, Germany; 3 Technical University Munich, IAS, CIPSM, Department of Chemistry, Garching, Germany; 4 Department Pharmazie Zentrum für Pharmaforschung, Ludwig-Maximilians-Universität München, Munich, Germany; Chiba University Center for Forensic Mental Health, JAPAN

## Abstract

The aim of this study was to design, synthesize and validate a multifunctional antidepressant probe that is modified at two distinct positions. The purpose of these modifications was to allow covalent linkage of the probe to interaction partners, and decoration of probe-target complexes with fluorescent reporter molecules. The strategy for the design of such a probe (i.e., azidobupramine) was guided by the need for the introduction of additional functional groups, conveying the required properties while keeping the additional moieties as small as possible. This should minimize the risk of changing antidepressant-like properties of the new probe azidobupramine. To control for this, we evaluated the binding parameters of azidobupramine to known target sites such as the transporters for serotonin (SERT), norepinephrine (NET), and dopamine (DAT). The binding affinities of azidobupramine to SERT, NET, and DAT were in the range of structurally related and clinically active antidepressants. Furthermore, we successfully visualized azidobupramine-SERT complexes not only in SERT-enriched protein material but also in living cells stably overexpressing SERT. To our knowledge, azidobupramine is the first structural analogue of a tricyclic antidepressant that can be covalently linked to target structures and further attached to reporter molecules while preserving antidepressant-like properties and avoiding radioactive isotopes.

## Introduction

Mapping monoamine transporters for relevant drug binding sites has been an integral part of elucidating the molecular mechanisms of antidepressants regarding their effects on the monoaminergic system. To achieve this, various experimental approaches have been pursued, including those employing chemically modified small molecules and genetic engineering. The chemically modified molecules used in these mapping studies typically consist of a pharmacologically active core structure enriched by a photo-inducible cross-linker and a radioactive isotope. This design allows the formation of compound-target complexes that are detectable by their radioactivity. In combination with genetic modifications of the target molecules, this approach enables the identification of functionally relevant amino acids of known targets. This strategy has successfully been used to characterize the binding sites of antidepressants to the monoamine transporters NET, DAT, and SERT [1–4]. Intriguingly, similar chemically modified tricyclic compounds (i.e. tritium labelled photo-labile tricyclic antidepressants) pointed to the existence of various binding partners in the cellular proteome that are most likely not identical to monoamine transporters [5–10]. However, not the least due to technical limitations at that time, the molecular identity of these candidates has never been revealed. Moreover, after the cloning of the monoamine transporters in the 1990s [11–13] the field focused mainly on these transporter molecules and (in-)directly associated pathways while neglecting potential alternative binding partners.

Today, several innovations in protein detection and chemical biology opened up hitherto unknown possibilities in molecular pharmacology. This is exemplified not only by phenotypic screening studies but also by the identification of direct interaction partners using multifunctional small molecules [14,15]. In particular, technical innovations in organic chemistry allowed the exchange of isotope labels by biologically inert chemical groups enabling for radioactive-free labeling of small molecule-target complexes. Despite promising results in other disciplines, no equivalent multifunctional tool derived from clinically approved antidepressants has been developed in the field of neuropsychopharmacology [16–21]. This may be due to the fact that mental diseases are multifactorial disorders with several layers of complexity and that antidepressant drugs are held to be promiscuous [22–25]. Moreover, like with other drug modifications, even small changes in chemical structure of psychoactive substances can result in considerable changes in target binding or even complete loss of activity [26].

The goal of this study was to modify an established antidepressant in a way that enables for covalent binding of the modified antidepressant to target structures and subsequent linkage of reporter molecules. We created azidobupramine, a structural analogue of imipramine, featuring two additional chemical groups, one for photoaffinity labelling (PAL) and the other for copper(I)-catalyzed azide alkyne cycloaddition (CuAAC). The former group allows for covalent linkage of azidobupramine to its target molecules and the latter to furnish the generated drug-target complexes with reporter molecules like fluorophores. For the biological evaluation of the functionality of azidobupramine, three canonical targets (i.e. SERT, NET and DAT) were used. Primary endpoints of the study were the analysis of binding affinities of azidobupramine to SERT, NET and DAT, and the functional evaluation of the added chemical moieties for PAL and CuAAC employing SERT as model target.

## Methods

### Chemical synthesis

Chromatographic separations were performed either by manual flash chromatography or by automated flash chromatography using an Interchim-Puriflash 430 with an UV detector. Organic phases were dried over MgSO_4_, and the solvents were removed under reduced pressure. Merck F-254 (thickness 0.25 mm) commercial plates were used for analytical TLC to follow the progress of reactions. Silica gel 60 (Merck 70-230 mesh) was used for manual column chromatography. Unless otherwise specified, ^1^H NMR spectra, ^13^C NMR spectra, 2D HSQC, HMBC and COSY of all intermediates were analyzed on a Bruker AC 300, a Bruker XL 400, or a Bruker AMX 600 at room temperature. Chemical shifts for ^1^H, ^13^C are given in ppm (δ) relative to tetramethylsilane (TMS) as internal standard. Mass spectra (m/z) were recorded on a Thermo Finnigan LCQ DECA XP Plus mass spectrometer, while the high resolution mass spectrometry was carried on Varian Mat711 mass spectrometer. The purity of the compounds was verified by reversed phase HPLC (Jupiter 4 μ Proteo 90 A, 250*4.6 mm, Phenomenex, Torrance, USA) using gradient A (acetonitrile: water gradient: 0.1% TFA of 0-100% in 45 min) unless otherwise specified. Solvents were purchased from Roth, reagents were obtained from Aldrich-Fluka unless otherwise noted. *HPLC conditions for product analysis; Column*: Jupiter 4 μm Proteo 90 A, 250 x 4.6 mm, Phenomenex, Torrance, USA, *Wavelength*: 224nm, 280nm *Flow rate*: 1ml/min, *Buffer A*: 0.1% TFA in 5% MeCN/Water, *Buffer B*: 0.1% TFA in 95% MeCN/water. *Gradient A* After 1min elution with 100% buffer A,: linear gradient of 0-100% buffer B for 30 min.

#### Synthesis of 1-(3-azido-10,11-dihydro-5H-dibenzo[b,f]azepin-5-yl)ethanone 8

To a stirred solution of 1-(3-amino-10,11-dihydro-5H-dibenzo [b,f]azepin-5-yl)ethanone **7** (*Wako Chemicals*, 100 mg, 0.396 mmol) in 10% aqueous hydrochloric acid (2ml), a solution of sodium nitrite (27.3 mg, 0.396 mmol) in water was added at 0–5°C with vigorous stirring. The mixture was kept below 5°C for 30 min, and then a solution of sodium azide (28.3 mg, 0.436 mmol) in water (5ml) was added dropwise while the reaction was kept at the same temperature. After being stirred for 1h, the mixture was warmed to room temperature and extracted with EtOAc and water. The organic layer was washed with brine, dried with MgSO_4_ and concentrated under reduced pressure to give 110mg (0.396 mmol, 100%) of compound **8** as yellow oily liquid. TLC (Hexane: EtOAc 11:9): R_f_ = 0.58. ^1^H NMR (400 MHz, CDCl_3_) δ = 2.03 (s, 3H, CH_3_), 2.77–2.86 (m, 2H, CH_2_CH_2_), 3.28–3.48 (m, 2H, CH_2_CH_2_), 6.85 (d, 1H, J = 8.4 Hz), 6.96 (s, 1H), 7.07 (s, 1H), 7.13 (d, 1H, J = 8.4 Hz), 7.29 (m, 4H). ^13^C NMR (100.5 MHz, CDCl_3_) δ = 22.64, 30.01, 30.59, 118.42, 119.19, 127.46, 127.60. 128.75, 129.88, 131.87, 137.41, 138.13, 140.94, 142.24, 143.69, 170.56. MS (ESI): m/z = 279.13 [M + H] ^+^, 301.13 [M+ Na] ^+^. Mass Calculated: 278.31.

#### Synthesis of 3-azido-10,11-dihydro-5H-dibenzo[b,f]azepine 9

To 110 mg of compound **8** (0.395 mmol) was added potassium hydroxide (72mg, 1.38 mmol) dissolved in 10ml of methanol and the reaction mixture was refluxed for 6 h under an argon atmosphere. Afterwards, methanol was evaporated and the mixture was extracted with CH_2_Cl_2_. The oily liquid was dissolved in minimum amount of EtOAc and then recrystallized from hexane in the cold to yield needle shaped crystals of **9** (85 mg, 0.359 mmol, 85%). TLC (Hexane: EtOAc 9:1): R_f_ = 0.6. ^1^H NMR (300 MHz, CDCl_3_) δ = 3.07 (s, 4H), 6.39 (d, 1H, J = 2.1 Hz), 6.49 (dd, 1H, J = 2.1, 6 Hz), 6.75 (d, 1H, J = 0.9 Hz), 6.84 (dt, 1H, J = 1.2, 7.5 Hz), 7.03 (d, 1H, J = 8.1Hz), 7.06–7.15 (m, 2H). ^13^C NMR (75 MHz, CDCl_3_) δ = 34.55, 34.81, 108.06, 109.83, 118.15, 120.12, 125.39, 126.95, 129.02, 130.64, 132.06, 138.55, 141.87, 143.53. MS (ESI): m/z = 237.20 [M + H] ^+^, Mass Calculated: 237.27 [M + H] ^+^.

#### Synthesis of 3-dimethylamino-1-propylchloride 10

Sodium hydroxide and 3-dimethylamino-1-propylchloride hydrochloride (TCI Europe) were dissolved separately in water (10 ml). These two solutions were mixed and the pH was adjusted to ~14. After extraction with dichloromethane (3x30 ml), the extracts were dried over anhydrous sodium sulfate and the solvent was removed to afford 50 mg (53%) of the free base. High vacuum was not used as the amine obtained is volatile.

#### Synthesis of 3-(3-azido-10,11-dihydro-5H-dibenzo[b,f]azepin-5-yl)-N,N-dimethyl propan-1-amine 11 (azidopramine)

A solution of compound **9** (50 mg, 0.212 mmol) was prepared in dry toluene (sure seal, Fluka, 10ml) at 0°C under argon. To the solution was added a suspension of NaH (6.09 mg, 0.254 mmol) in toluene (3ml) and the reaction was stirred for 30min. Freshly prepared solution of 3-dimethylamino-1-propylchloride **10** (33.5mg, 0.275 mmol) (generated from its hydrochloride salt) as described above was added dropwise and the reaction was allowed to warm to room temperature. The reaction was heated to 60°C and stirred overnight. TLC analysis showed complete disappearance of the starting educt **9**. The reaction mixture was poured into water and extracted with EtOAc. The organic layer was washed with brine, dried over MgSO_4_ and concentrated by rotary evaporation. Column chromatography in dichlorormethane: MeOH 95:05 of the crude reaction mixture was performed to give compound **11** (azidopramine, 45 mg, 0.14mmol, 66%). TLC (DCM: MeOH 9:1): R_f_ = 0.38. HPLC (gradient A) Rt: 21.2min, Purity = 99%. ^1^H NMR (300 MHz, CDCl_3_) δ = 1.70–1.80 (m, 2H), 2.19 (s, 6H), 2.26–2.37 (m, 2H), 3.15 (s, 4H), 3.76 (t, 2H, J = 6.9 Hz) 6.61 (dd, 1H J = 2.4, 5.7 Hz), 6.73 (d, 1H, J = 2.1 Hz), 6.984 (dt, 1H, J = 1.5, 5.4, 6.9 Hz), 7.06 (d, 1H, J = 8.1Hz), 7.11–7.20 (m, 3H). ^13^C NMR (75 MHz, CDCl_3_) δ = 25.94, 31.65, 32.22, 45.42, 48.84, 57.49, 110.46, 112.45, 120.56, 123.22, 126.55, 129.43, 129.96, 131.33, 135.17, 137.87, 147.91, 149.20. MS (ESI): m/z = 322.27 [M + H] ^+^. HRMS: 322.1861[M + H] ^+^, Mass Calculated: 322.1853 [M + H] ^+^.

#### Synthesis of 3-(tert-butoxycarbonyl(methyl)amino)propyl 4-methylbenzenesulfonate 13

To 3-(methylamino)propan-1-ol **12** (250 mg, 6.28 mmol) in acetonitrile was added BOC anhydride (680 mg, 12.56 mmol) and a catalytic amount of DMAP. The reaction was stirred for 2 hours until the disappearance of alcohol **12**. The crude mixture was subjected to column chromatography (Hexane: EtOAc 55:45) and dried under reduced pressure to obtain (460 mg, 2.43 mmol, 87%) of the desired product. TLC (Hexane: EtOAc 1:1): R_f_ = 0.46. ^1^H NMR (300 MHz, CDCl_3_) δ = 1.45 (s, 9H), 1.66–167 (m, 2H), 2.62 (s, 3H), 3.37 (s, 2H), 3.52 (s, 2H). ^13^C NMR (75 MHz, CDCl_3_) δ = 28.35, 29.63, 34.13, 44.21, 58.08, 79.96, 157.19.

To the above compound (90 mg, 0.475 mmol) in dichloromethane was added p-toluene sulfonylchloride (136 mg, 0.713 mmol) and triethylamine (96 mg, 0.951 mmol) and the mixture was stirred at 0°C for 4 h. The reaction mixture was then quenched with water and extracted using diethyl ether. The ethereal layer was washed with brine and dried over MgSO_4_ to yield the crude product which was further subjected to column chromatography using Hexane: EtOAc 13: 7 to yield 135 mg (0.393 mmol, 84%) of **13** as white oily liquid. TLC (Hexane: EtOAc 1:1): R_f_ = 0.46. ^1^H NMR (300 MHz, CDCl_3_) δ = 1.41 (s, 9H), 1.81–1.90 (m, 2H), 2.44 (s, 3H), 2.78 (s, 3H), 3.23 (t, 2H, J = 6.9 Hz), 4.02 (t, 2H, J = 6.3 Hz), 7.34 (d, 2H, J = 7.8 Hz), 7.77 (d, 2H, J = 8.4 Hz). ^13^C NMR (75 MHz, CDCl_3_) δ = 21.63, 27.53, 28.35, 34.59, 45.28, 68.2, 79.62, 125.94, 127.87, 129.10, 129.88, 132.86, 144.87, 155.60. MS (ESI): m/z = 244.13 [M—Boc] ^+^, Mass Calculated: 244.08[M—Boc] ^+^.

#### Synthesis of tert-butyl 3-(3-azido-10,11-dihydro-5H-dibenzo[b,f]azepin-5-yl)propyl(methyl) caRbBAmate 14

To a solution of 3-azido-10,11-dihydro-5H-dibenzo[b,f]azepine **9** (130 mg, 0.550 mmol) in 5ml dry toluene was added 0.660ml of a 1M solution of sodium bis (trimethylsilyl) amide in hexane (0.660 mmol) under an argon atmosphere at -78°C and the mixture was stirred for 0.5h. Freshly prepared tosyl analog **13** (227 mg, 0.660 mmol) was added dropwise to the above mixture and the reaction flask was allowed to warm to room temperature. The reaction was further stirred at 70°C overnight and the completion of the reaction was monitored using thin layer chromatography. The crude product was poured into water and extracted with EtOAc. The organic layer was washed with brine and dried over MgSO_4_ and concentrated to dryness. Column chromatography of the crude reaction mixture was performed in (Hexane: EtOAc 19:1) as eluent to give compound **14** (150 mg, 0.368 mmol, 67%). TLC (Hexane: EtOAc 19:1): R_f_ = 0.23. ^1^H NMR (400 MHz, CDCl_3_) δ = 1.39 (s, 9H), 1.72–1.79 (m, 2H), 2.44 (s, 3H), 2.72 (s, 3H), 3.09–3.16 (m, 4H), 3.23 (t, 2H, J = 6.8 Hz), 3.69 (t, 2H, J = 6.8 Hz), 6.59 (dd, 1H, J = 2.4, 5.6 Hz), 6.68 (d, 1H, J = 2 Hz), 6.96 (dt, 1H, J = 1.2, 7.2 Hz), 7.05 (t, 2H, J = 8 Hz), 7.10–7.16 (m, 2H). ^13^C NMR (100 MHz, CDCl_3_) δ = 26.26, 28.39, 31.58, 32.14, 34.23, 46.71, 48.09, 79.31, 110.27, 112.52, 120.36, 123.33, 126.54, 129.49, 129.91, 131.37, 135.17, 137.89, 147.67, 149.12, 155.69.

#### Synthesis of 3-(3-azido-10,11-dihydro-5H-dibenzo[b,f]azepin-5-yl)-N-methylpro-pan-1-amine 15 (desazidopramine)

Compound **14** (150mg, 0.368mmol) was deprotected in the presence of 20% trifluoroacetic acid solution in DCM for 3.5h at room temperature to yield **15**. TFA was evaporated under reduced pressure and the crude mixture was subjected to a small wash out silica gel column using hexane: EtOAc: TEA 3.8:6.0:0.2 to give pure desazidopramine **15** (85mg, 0.276mmol, 75%). TLC (Hexane: EtOAc: TEA 3.8:6.0:0.2): R_f_ = 0.29. HPLC (gradient A) Rt: 19.2 min, Purity = 98% ^1^H NMR (600 MHz, CDCl_3_) δ = 1.88–1.93 (m, 2H), 2.42 (s, 3H), 2.85 (t, 2H, J = 7.2), 3.07–3.12 (m, 4H), 3.75 (t, 2H, J = 6.6 Hz), 6.62 (dd, 1H, J = 2.4, 6 Hz), 6.64 (d, 1H, J = 1.8 Hz), 6.967(dt, 1H, J = 1.2, 6 Hz), 7.03 (q, 2H, J = 4.8, 7.2 Hz), 7.10–7.15 (m, 2H). ^13^C NMR (150 MHz, CDCl_3_) δ = 24.69, 31.48, 31.96, 33.26, 47.49, 47.62, 110.15, 112.95, 120.12, 123.71, 126.70, 129.68, 129.98, 131.50, 135.06, 138.08, 147.15, 148.68. MS (ESI) m/z 308.12 [M + H] ^+^. HRMS: 308.1685 [M + H] ^+^, Mass Calculated: 308.1704 [M + H] ^+^.

#### Synthesis of N-(3-(3-azido-10,11-dihydro-5H-dibenzo[b,f]azepin-5-yl)propyl)-N-methylbut-3-yn-1-amine 17 (azidobupramine)

To a solution of **15** (80 mg, 0.260 mmol) in acetone (10ml) was added potassium carbonate (180mg, 1.30 mmol) and a catalytical amount of potassium iodide. The mixture was stirred for 30 min and then further reacted with the 4-bromobut-1-yne **16** (41mg, 0.312 mmol) and refluxed at 60°C overnight. Acetone was evaporated followed by an aqueous work up and extraction with CH_2_Cl_2_. The crude mixture was subjected to column chromatography in DCM: MeOH mixture to give the desired product **17** (47 mg, 0.130 mmol, 50%). TLC (DCM: MeOH 9.2:0.8): R_f_ = 0.38. HPLC (gradient A) Rt: 19.8 min, Purity = 85% ^1^H NMR (300 MHz, CDCl_3_) δ = 1.69–1.79 (m, 2H), 1.94–1.96 (t, 1H, J = 2.4 Hz), 2.21 (s, 3H), 2.27–2.33 (m, 2H), 2.45 (t, 2H, J = 7.5 Hz), 2.56 (t, 2H, J = 7.2 Hz), 3.15 (s, 4H), 3.78 (t, 2H, J = 6.9 Hz), 6.62 (dd, 1H, J = 2.4 Hz), 6.74 (d, 1H, J = 2.4 Hz), 6.90–7.20 (m, 5H). ^13^C NMR (75 MHz, CDCl_3_) δ = 15.73, 24.45, 30.55, 31.33, 40.86, 47.62, 53.77, 54.90, 67.86, 81.68, 109.36, 111.29, 119.45, 122.09, 125.41, 128.30, 128.81, 130.21, 134.06, 136.75, 146.80, 148.10. MS (ESI): m/z = 360.16 [M + H] ^+^. HRMS: 360.2057 [M + H] ^+^, Mass Calculated: 360.2061[M + H] ^+^.

### Biological evaluation

#### Materials/Chemicals

The following materials were purchased: Dulbecco’s Modified Eagle’s Medium (DMEM), FreeStyleTM 293 Expression medium, Fetal Bovine Serum (FBS), antibiotic-antimycotic, streptomycine, sodium pyruvate, and tetracycline (*Life Technologies*, *CA*, *USA*); blasticidin and zeocin (*InvivoGen*, *CA*, *USA*); citalopram, lofepramine, and paroxetine (*Kemprotec Limited*, *Middlesbrough*, *UK)*; desipramine, imipramine, polyethyleneimine, ANTI-FLAG^®^ M2 Affinity Gel, SIGMAFASTTM Protease Inhibitor Cocktail EDTA-free (*SigmaAldrich*, *MO*, *USA*); [^3^H]-citalopram (84 Ci/mmol), clear 96-well Flexible PET Microplate (*PerkinElmer LAS*, *Boston*, *MA*, *USA)*; Rotiszint^®^ eco plus scintillant (*Carl Roth*, *Karlsruhe*, *Germany)*.

#### Cell culture

T-REx-SERT cells expressing rSERT-His10-FLAG were kindly provided by C.G. Tate (*MRC Laboratory of Molecular Biology*, *Cambridge*, *UK*) [27]; cells were cultivated in suspension (FreeStyle^™^ 293 Expression medium, 10% FBS, antibiotic-antimycotic, and pyruvate); for selection purpose, 5 μg/ml blasticidin and 200 μg/ml zeocin were used; expression was induced by adding 1 μg/ml tetracycline for 5 days.

#### Membrane preparation

Tetracycline induced T-REx-SERT cells were mechanically disrupted with a hypotonic Tris-buffer (50 mM Tris, pH 7.9, proteinase inhibitor) in a two step procedure: dounce-homogenisation (*Potter S*, *B*.*Braun*, *Melsungen*, *Germany)* followed by an ultrasound treatment (*sonifier W-250*, *Branson*, *CT*, *USA*). Homogenates were centrifuged first at 800 rcf (10’, 4°C), followed by a second centrifugation step at 100,000 rcf (60’, 4°C). The resulting pellet was re-suspended in reaction-buffer RB1 (50 mM Tris-HCl, 150 mM NaCl, 5 mM KCl, proteinase inhibitor, pH 7.9), and stored at -80°C after protein determination (*Pierce*^®^ *BCA Protein Assay*, *Thermo Scientific*, *IL*, *USA*).

#### Analysis of substance 17 for its binding to SERT, NET, and DAT: Mass-Spectrometry Based Assay (MSBA)

Competitive MSBAs to characterize the affinity of test compounds employing (1*R*,3*S*)-indatraline as a marker for hDAT, hNET and hSERT, respectively, were performed exactly as described by Grimm et al. [28]. For this purpose, membrane fractions obtained from HEK293 cell lines stably expressing the corresponding target protein were incubated in presence of a fixed concentration of (1*R*,3*S*)-indatraline together with varying concentrations of test compounds in 96 well plates. After separation of the non-bound marker by vacuum filtration from the binding samples, bound (1*R*,3*S*)-indatraline remaining on the filter was eluted with acetonitrile (containing internal standard) and quantified by LC-ESI-MS/MS. Subsequently, inhibition of (1*R*,3*S*)-indatraline binding caused by test compounds could be analyzed essentially in the same way as described for radioligand binding assays.

#### Analysis of substances 17, 15, and 11 for their binding to rSERT: Radioligand Binding Assays (RBA)

Protocols for radioligand binding assays (RBA) were adapted from Basile (saturation experiments) and Nakaki (competition experiments) [29,30]. Briefly, reactions were carried out in reaction-buffer RB1 containing 1 μg protein of the membrane preparation overexpressing rSERT, using various concentrations of [^3^H]-citalopram and, depending on the assay, different competitors. Drugs were diluted in DMSO as solvent. Incubation was carried out at 34°C for 60’, and terminated by rapid filtration over polyethyleneimine preincubated GF/C filters using a Brandell MWXR-97TI cell harvester (*Gaithersburg*, *MD*, *USA*). Filters were washed three times with 2 ml of ice cold reaction-buffer RB1 and then incubated with scintillation liquid; radioactivity was measured by beta counting (*MicroBeta*, *PerkinElmer LAS*, *Boston*, *MA*, *USA*). Maximal binding sites (B_max_) and equilibrium affinity constants (*K*_d_) were determined using [^3^H]-citalopram as radioligand in concentrations ranging from 0.09 nM to 60 nM; nonspecific binding was determined in the presence of 50 μM imipramine. For competition experiments, the concentration of [^3^H]-citalopram was set to 0.5 nM; each competitor was titrated in ten dilution steps ranging from 1 fM to 30 μM; maximal radioligand binding was determined in the absence of any competitor, and nonspecific binding in the presence of 50 μM imipramine.

#### Analysis of 17 for photoaffinity labeling (PAL), and Copper(I)-catalyzed azide alkyne cycloaddition (CuAAC): fluorescence based assays

In addition to the determination of binding affinities of azidobupramine (**17**) to hSERT, hNET and hDAT, also the functionality of the added chemical groups of **17**, namely photoaffinity labeling (PAL) and copper(I)-catalyzed azide alkyne cycloaddition (CuAAC), were evaluated in different biological systems. For all these experiments, rSERT was chosen as model target. The PAL-reaction was conducted either using rSERT-enriched protein material or living cells. In both cases, the subsequent CuAAC-reaction took place after the double-tagged rSERT-His10-FLAG was immobilized on ANTI-FLAG^®^ M2 Affinity-gel (*SigmaAldrich*, *MO*, *USA*).

For the interaction analysis of azidobupramine (**17**) with rSERT-enriched material, 1 mg of membrane preparation was solubilized in reaction-buffer RB3 (52.6 mM Tris/HCl, 126.4 mM NaCl, 5.26 mM KCl, 1% Triton X-100, proteinase inhibitor, pH 7.9). Then rSERT was immobilized on ANTI-FLAG^®^ M2 Affinity-gel (over night, 4°C, constant agitation), and unbound protein was removed by three washing steps with reaction-buffer RB4 (25 mM HEPES/NaOH, 126.4 mM NaCl, 5.26 mM KCl, proteinase inhibitor, pH 7.9). Depending on the condition to be tested, **17** (1 μM) alone or in the presence of 1000 fold molar excess (1 mM) of two different competitors (i.e. paroxetine, mirtazapine) were added to the reaction (90’, RT, constant agitation). This was followed by UV-light exposure (312 nm, 2x 45”, RT) using the Dual Transilluminator from Stratagene (5x8 Watt, *La Jolla*, *CA*, *USA*), and copper mediated click reaction (60 μM Rhodamine-azide, 2.5 mM ascorbic acid, 250 μM CuSO4, and 500 μM bis[(tertbutyltriazoyl)methyl]-[(2-carboxymethyltriazoyl)methyl]amine (BTTAA), 60’, RT, constant agitation) [31]. Finally, rSERT was eluted from affinity gel with Laemmli buffer (65°C, 15’), and separated by SDS-PAGE (12%).

For the interaction analysis in living cells, 42 million suspended T-REx-SERT cells in 2ml cultivation medium, overexpressing rSERT, were incubated with **17** (10 μM) alone or in the presence of equimolar concentrations of paroxetine at constant agitation for 30’ (37°C, 5% CO_2_). This was followed by UV-light induced covalent linkage (312 nm, 2x 60”, RT). The further processing was as described above, i.e. membrane preparation, solubilization and immobilization of rSERT to ANTI-FLAG^®^ M2 Affinity-gel, CuAAC-reaction followed by protein elution, and SDS-PAGE.

#### Data analysis

Bmax and *K*d values were determined by means of non-linear regression analysis using SigmaPlot 11 (*Systat Software Inc*., *IL*, *USA*); applied algorithm: one side saturation. pIC_50_ values were determined by means of non-linear regression analysis using SigmaPlot 11; applied algorithm: sigmoidal dose response; bottom (nonspecific binding) and top (no competition) of the curves were set to 0 and 1, corresponding to 0% and 100% respectively.

Within the fluorescence based interaction studies of **17** with rSERT, the fluorescence signal was recorded with the *ChemiDoc MP detection-system* and analyzed using ImageLab (*BioRad*, *CA*, *USA*); for quantification, only those fluorescent signals were taken that correspond to Western blot verified rSERT (between 70-100 kDa). Recorded fluorescent signals were normalized to the Western-blot signal of rSERT. Statistical evaluation was performed using either the Student t-Test (normal distributed sample, two independent groups), the Wilcoxon-Mann-Whitney-Test (non-normal distributed sample, two independent groups), or ANOVA (normal distributed sample; more than two groups); for post-hoc analyses, most stringent tests were used (indicated in each analysis). The levels of significance were *p < 0.05, **p < 0.01, and ***p < 0.001, respectively.

## Results and Discussion

### Design and synthesis of azidobupramine (17)

**Fig 1:**
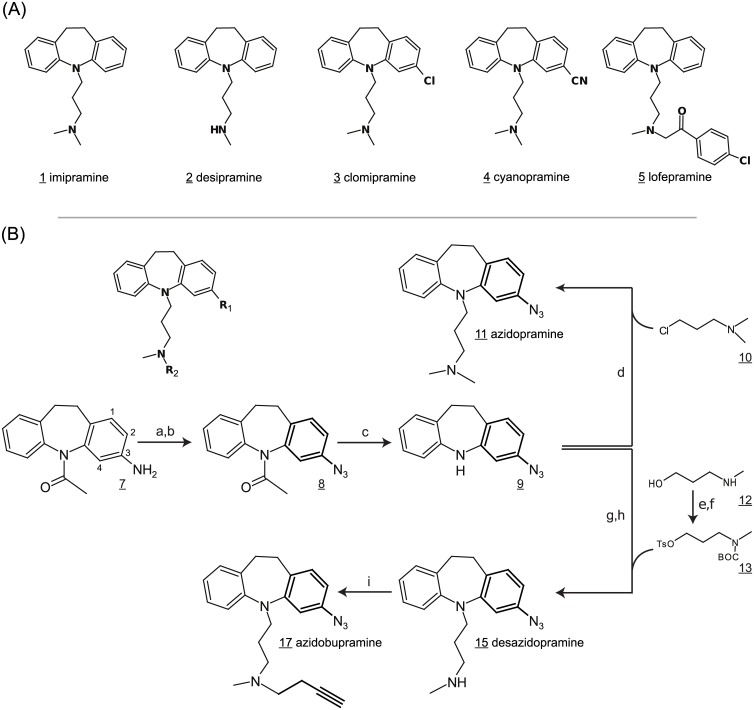
Synthesis scheme of multifunctional antidepressants and reference compounds. (**A**) Reference substances: overview of clinically active antidepressants (**1**, **2**, **3**, **4**, **5**); (**B**) Synthesis scheme of azidobupramine: *a)* NaN_3_, H_2_O, RT, 1h. *b)* NaNO_2_, 10% HCl *c)* KOH, MeOH, 60°C, 15h. *d)* NaH, ClCH_2_CH_2_CH_2_N(CH_3_)_2_ (10), 0–60°C, toluene. *e)* (Boc)_2_O, DMAP, ACN, RT, 2h. f) TsCl, Et3N, DCM, 0°C. *g)* NaHMDS, toluene, -78°C to 70°C, 6h. *h)* 20% TFA, DCM, RT, 4h. *i)* 1-bromo-3-butyne, K_2_CO_3_, KI, 60°C, 12h, acetone.

For the synthesis of azidobupramine (**17**), we began with the prototypic tricyclic antidepressant imipramine (**1**) as a chemical starting point. Previous structure activity relationship studies (SARS) on tricyclic antidepressants and recent cocrystal structures of antidepressant-transporter complexes indicated that substituents at position three of the tricyclic ring system and at the terminal amine of imipramine could be tolerated by classical monoamine transporters [32–43]. Furthermore, clinically effective analogues like desipramine (**2**), clomipramine (**3**), cyanopramine (**4**) and lofepramine (**5**) suggested that substituents at the ring structure and at the terminal amino group do not compromise antidepressant activity on the clinical level (Fig 1A). We thus set out to introduce an azido group at position 3 of imipramine (**1**) to graft the known photoreactivity of aromatic azides into the tricyclic ring system. The alkyne tag for CuAAC was designed to be introduced at the terminal amino function.

For the chemical synthesis of the multifunctional tricyclic antidepressant analogues azidopra-mine (**11**), azidodesipramine (**15**), and azidobupramine (**17**), a common building block **9** was synthesized from the commercially available azepine analogue 1-(3-amino-10,11-dihydro-5H-dibenzo[b,f]azepin-5-yl)ethanone (**7**). The primary amine was first converted to the corresponding azide **8** followed by base-assisted deprotection of the amino group and subsequent alkylation with **10** to yield the azidopramine (**11**) (Fig 1B). Azidobupramine (**17**) was synthesized via the intermediate product azidodesipramine (**15**). The coupling of the necessary Boc-protected building block **13** to azepine **9** turned out to be more demanding than that of the corresponding (3-chloropropyl) dimethylamine **10**, which was used for the synthesis of **11**. Stronger activation with a tosyl group and optimization of the reaction conditions eventually yielded compound **15**; a further alkylation step finally yielded the desired fully equipped azidobupramine (**17**) (Fig 1B).

### Analysis of binding properties to monoamine transporters (SERT, NET, and DAT)

After the synthesis, it was necessary to check whether the two functional groups incorporated into azidobupramine (**17**) may have changed known antidepressant-like pharmacological properties inherent to the parent molecule. Several biochemical, cell- and animal-based test systems have been developed to evaluate small molecules for their antidepressant-like pharmacological properties. While none of these tests alone or in combination can replace clinical trials as ultimate test, analyzing binding properties of small molecules to monoamine transporters is a frequently and successfully used approach to predict the likelihood of the potential of these molecules to act as antidepressants.

To define binding parameters of azidobupramine (**17**) to the monoamine transporters two complementary techniques were applied, the MS based Binding Assay (MSBA) and the classical Radioligand Binding Assay (RBA).

**Fig 2:**
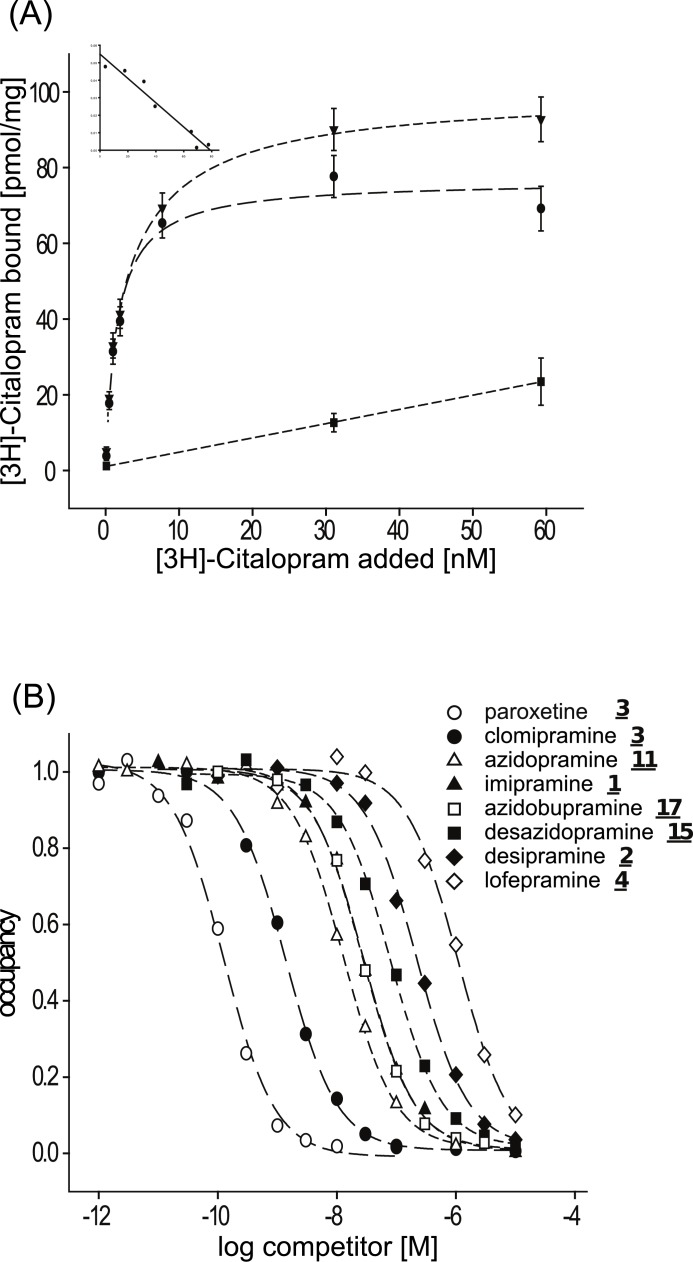
Binding parameters of 17 and reference compounds for rSERT. (**A**) Saturation analysis using 1 μg membrane homogenate per reaction from recombinant rSERT expressing cells with [^3^H]-citalopram as radio-ligand; the maximal number of binding sites (Bmax±SEM) and equilibrium dissociation constants for the radioligand (*K*d±SEM) were calculated by means of non-linear regression analysis (Bmax 77.2±1.8 pmol/mg; *K*d 1.8±0.2 nM) and verified after Scatchard transformation (inset: Bmax 80.3±1.5 pmol/mg; *K*d 2.1±0.4 nM); each data point represents the average value (±SEM) of 12 independent experiments. (**B**) 0.5 nM [^3^H]-citalopram was used for competition experiments; dilutions were prepared from **17**, **15**, **11**, and paroxetine, clomipramine, imipramine, desipramine and lofepramine; each data point represents the average value of at least eight independent experiments.

MSBA revealed that azidobupramine (**17**) is characterized by moderate to high affinities to hSERT, hNET, and hDAT leading to a *K*_i_-value-based ratio of 1:1:16 (Table 1). RBA with rSERT as target structure indicated that binding affinities of azidobupramine (**17**) and two related compounds (i.e. **15**, **11)** follow a well-known structure dependent pattern. This includes the relation to five clinically active substances (Table 2; Fig 2A and 2B). While the substitution of position 3 at the cyclic head-structure with an azido group consistently leads to higher affinities (**1** *K*_i_ = 17.5 nM, **11** *K*_i_ = 8.82 nM, **2** *K*_i_ = 217 nM and **15** *K*_i_ = 62.4 nM), variations at the terminal amino group result in divergent effects. Secondary amino functions at the terminal amino group go along with lower affinities (**15**: *K*_i_ = 62.4 nM, **2**: *K*_i_ = 217 nM) and tertiary amino functions with higher affinities (**11**: *K*_i_ = 8.82 nM, **1**: *K*_i_ = 17.5 nM). Furthermore, it appears evident that *K*_i_ values do not only depend on the type of the terminal amino group (secondary versus tertiary) but also on the size and structure of the attached group—the more sterically demanding the chemical group, the larger the influence on the binding parameters [32,37]. This influence of the substitution at the terminal amino group on binding affinities to rSERT can be illustrated by comparing the *K*_i_-values of imipramine (**1**) (*K*_i_ = 17.5 nM) and lofepramine (**5**) (*K*_i_ = 1.12*10^3^ nM) and two newly synthetized ring substituted derivatives **11** (*K*_i_ = 8.82 nM) and **17** (*K*_i_ = 22.2 nM).

**Table 1:** Equilibrium affinity constants pK_i_ ±SEM for MSBA and pIC_50_ ±SEM for RBA and corresponding binding affinities (K_i_) of 17 to SERT, NET, and DAT; affinity constants were measured using two different technical approaches based on mass spectrometry (MSBA), and based on classical radioligand binding assays (RBA); each data point represents the average value of three independent experiments, each performed in triplicates.

Method	SERT		NET		DAT	
	pK_i_/pIC_50_	K_i_ [nM]	pK_i_/pIC_50_	K_i_ [nM]	pK_i_/pIC_50_	K_i_ [nM]
MSBA	6.99±0.03	103	6.59±0.06	115	5.80±0.07	1.64*10^3^
RBA	7.52±0.03	22.2	----	----	----	----

**Table 2:** Equilibrium affinity constants (pIC_50_ ±SEM) and corresponding binding affinities (K_i_) of 17, 15, 11, and the antidepressants paroxetine, imipramine (1), desipramine (2), clomipramine (3) and lofepramine (5) at rSERT; the conversion of IC_50_ to *K*_i_ values was carried out according to Cheng-Prusoff while using the actual [^3^H]-citalopram concentration for each calculation [44]; each data point represents the average value of at least eight independent experiments.

Compound	Acronym	pIC_50_±SEM	K_i_ [nM]
Non-Tricyclic Antidepressants			
Paroxetine	PAR	10.02±0.07	0.07
Tricyclic Antidepressants (unmodified)			
Clomipramine	CMI	8.88±0.07	0.97
Imipramine	IMI	7.63±0.04	17.5
Desipramine	DMI	6.53±0.06	217
Lofepramine	LOF	5.81±0.08	1.12*10^3^
Analogues of Tricyclic Antidepressants			
Azidopramine	11	7.92±0.04	8.82
Azidobupramine	17	7.52±0.03	22.2
Desazidobupramine	15	7.07±0.03	62.4

The binding affinities of azidobupramine (**17**) to SERT determined by RBA and MSBA differ to some degree. While the radioactivity-based assay revealed a *K*_*i*_-value of 22.2 nM, there was a 4.6-fold drop in affinity using the mass-spectrometry-based assay (103 nM). This difference might be due to the origin of the protein source (rat for RBA, human for MSBA), variations in the harvesting procedure and buffer conditions, and different reporter ligands used (citalopram for RBA, indatraline for MSBA). Although the reason for these differences remains to be investigated, we would like to point out that the magnitude of the difference between these two methods is not unusual [28]. In addition, the *K*_*i*_-values determined here with each method are well in the range of data reported in the literature [45,46].

### Analysis of 17 for photoaffinity labelling (PAL), and copper(I)-catalyzed azide alkyne cycloaddition (CuAAC)

Having proven that azidobupramine (**17**) is characterized by high affinity to the monoamine transporters, we next tested the functionality of the two additional groups of azidobupramine (**17**): UV-light induced photoaffinity labelling (PAL) by the aryl-azide group and copper(I)-catalyzed azide alkyne cycloaddition (CuAAC) by the terminal alkyne group. Specifically, we evaluated whether azidobupramine (**17**) can be covalently linked to rSERT by UV-light (PAL) and subsequently be furnished with fluorochromes by CuAAC for visualization.

**Fig 3:**
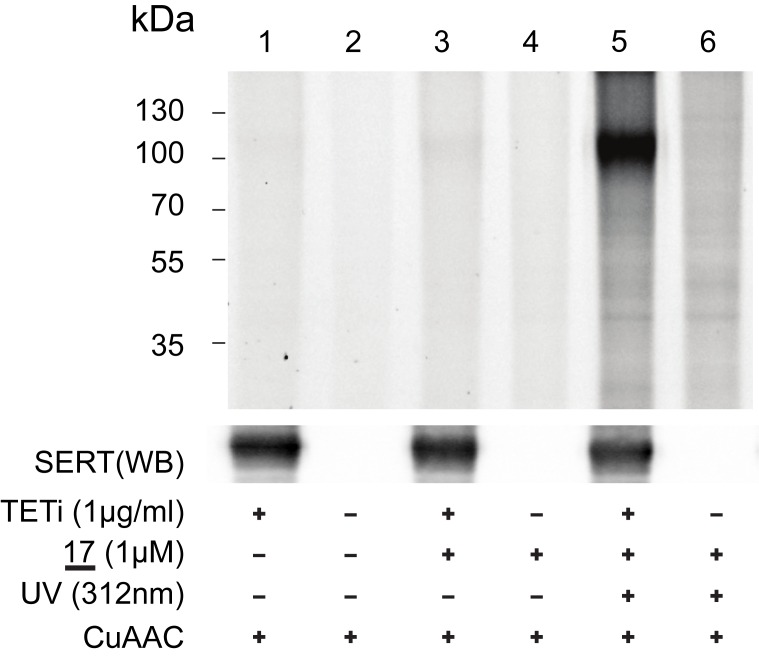
Analysis of 17 for PAL and CuAAC. Membrane fractions were prepared from tetracycline induced (TETi) and non-induced T-REx-SERT cells; differential expression of rSERT was verified by Western blot analysis (lower panel); while the CuAAC-mediated rhodamine-labelling reaction was carried out in all conditions, **17**-rSERT complexes are only visualized if rSERT is expressed, **17** is present and the reaction was exposed to UV-light (upper panel, lane5); the blot displayed is a representative example of five independent experiments. *The full*, *uncropped original images of the fluorescence and Western blot analyses are provided in* S1 *and* S2 Figs.

In a first step, it was necessary to demonstrate that azidobupramine (**17**) forms covalent complexes with rSERT dependent on the expression status of rSERT, the PAL-reaction, and the CuAAC-reaction. To this end, membrane preparations from rSERT overexpressing and control cells were solubilized and loaded onto ANTI-FLAG^®^ M2 Affinity gel matrix. The experiments were designed in such a way that visualization of rSERT by fluorescence is only possible if three conditions are fulfilled: presence of rSERT, functionality of the incorporated chemical moieties into (**17**) for PAL and CuAAC. In fact, the specific fluorescent signal became apparent only when rSERT was expressed, azidobupramine (**17**) was added to the reaction, and the material was exposed to UV-light (Fig 3, lane 5). We conclude that all functional features of azidobupramine (**17**) are operative and that **17** interacts predominantly with rSERT after the transporter was enriched onto ANTI-FLAG^®^ M2 Affinity gel.

**Fig 4:**
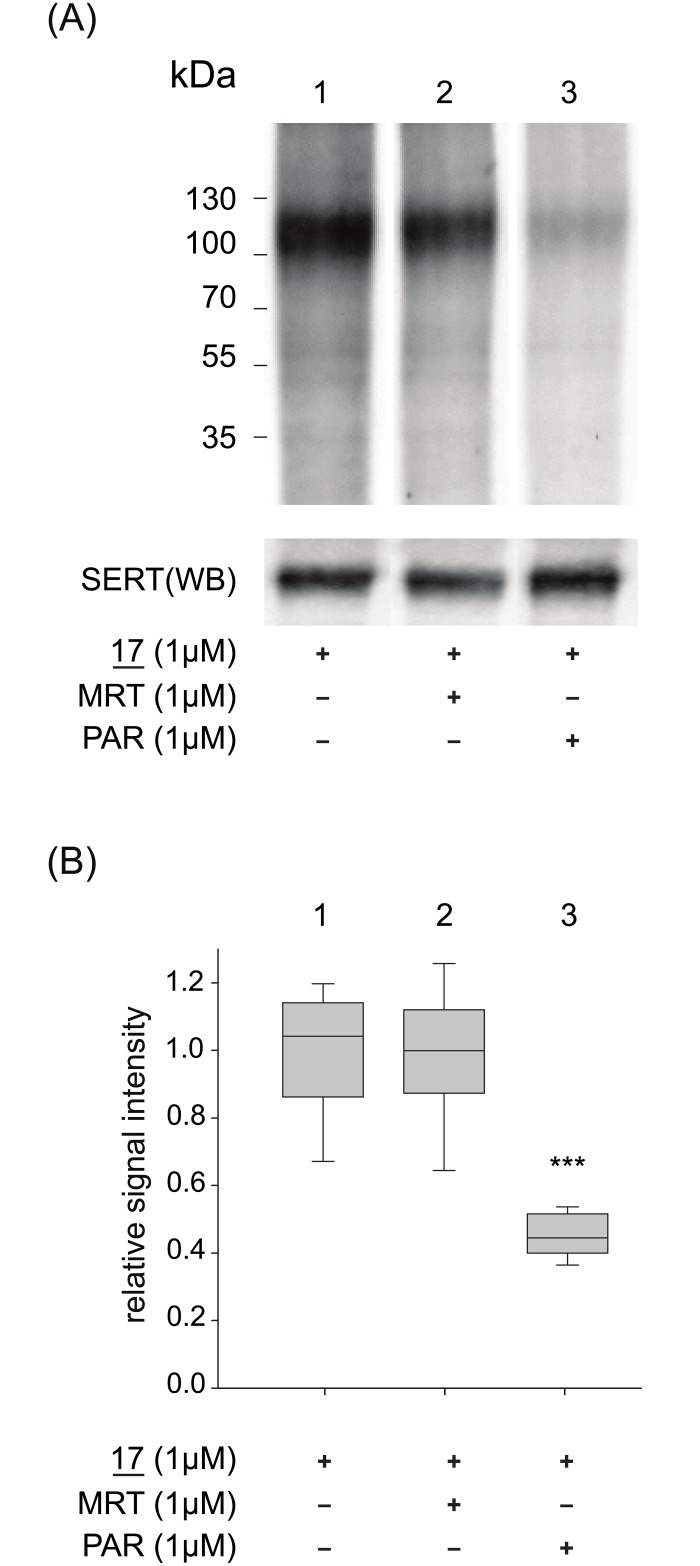
Competition experiments of azidobupramine (17) binding at enriched rSERT by paroxetine (PAR) and mirtazapine (MRT). Membrane preparations from rSERT expressing cells were incubated with **17** in the absence or presence of PAR or MRT. (**A**) After PAL and CuAAC-mediated fluorescent labelling, proteins were separated by SDS-PAGE and fluorescence was monitored; expression of rSERT was verified by Western blot analysis (lower panel). (**B**) PAR leads to a 60% decrease in binding signal (***p < 0.001) while MRT has no effect; for data analysis one way ANOVA followed by Bonferroni adjustment was applied; each box-plot represents the average of 12 independent experiments, each performed in triplicates.

To further elaborate the interaction of azidobupramine (**17**) and rSERT, competition experiments were designed in the presence of either paroxetine, which is characterized by high binding affinity to SERT (Fig 4 lane 3) or mirtazapine, which shows no relevant binding to the transporter (Fig 4 lane 2). As in the experiments before, the binding reactions were performed after rSERT was immobilized on ANTI-FLAG^®^ M2 Affinity gel. It was apparent, that only paroxetine significantly displaced azidobupramine (**17**) from rSERT while mirtazapine does not. Reasons for the incomplete displacement of azidobupramine (**17**) from rSERT (i.e. 60%) could be attributed to the hypothesized additional low affinity binding sites of tricyclic antidepressants at the monoamine transporters [5,47] that could hardly be monitored by conventional binding assays such as RBA or MSBA. Alternatively, we cannot exclude that detergents alter membrane proteins in their secondary and tertiary structure leading to the presentation of unspecific binding sites [48]. Binding of azidobupramine (**17**) to such sites would be less likely outcompeted by paroxetine. Based on this, we hypothesize that the 60% decrease of signal intensity corresponds to the canonical and intact binding sites of antidepressants to the transporter molecules, while the remaining 40% represents the non-specific interaction of azidobupramine (**17**) with rSERT, be it the proposed additional binding sites for tricyclic antidepressants or hydrophobic surfaces exposed by partial denaturation.

**Fig 5:**
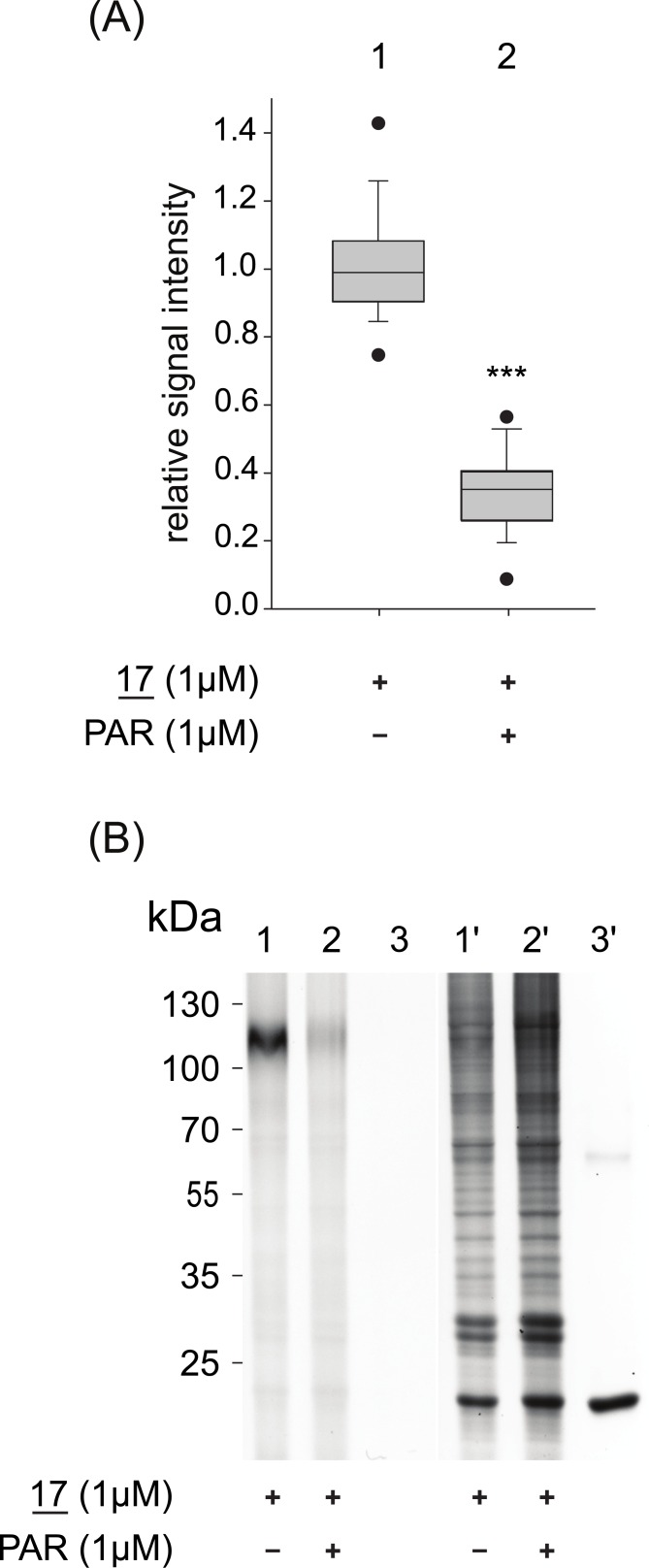
Analysis of azidobupramine (17) binding to rSERT in living cells. Living rSERT expressing cells were exposed to **17**, in the presence or absence of PAR followed by PAL-reaction, still in living cells; immediately afterwards we performed cell disruption, membrane purification, affinity enrichment and CuAAC-mediated rhodamin labelling. (**A**) Relative change of **17**-specific binding to rSERT depending on the presence of the competitor PAR; Wilcoxon-Mann-Whitney test was applied, ***p <0.001; each box-plot represents the average of 20 independent experiments, each performed in triplicates. (**B**) Representative fluorescence scans (lanes 1–3) and corresponding Coomassie-stained gels (lanes 1’-3’); lanes 3 and 3’ show control eluates from affinity gels that were not loaded with protein material.

To control for possibly detrimental detergent mediated effects on protein structures, the interaction of azidobupramine (**17**) with rSERT was also examined in living cells (Fig 5). Azidobupramine (**17**) was allowed to form complexes with rSERT under cell culture conditions (including PAL), while the indicator CuAAC reaction took place after rSERT was enriched on ANTI-FLAG^®^ M2 Affinity gel. We could demonstrate that azidobupramine (**17**) also forms covalent bonds with rSERT after UV-light exposure in living cells. In competition experiments using an equimolar amount of paroxetine (10μM), a decrease of fluorescent signal of 60–70% was observed (Fig 5, compare lanes 1 and 2 in panel B, quantification in panel A). Considering the higher affinity of the competitor paroxetine in comparison to azidobupramine (**17**) (Table 2), one might expect higher displacement of **17** by paroxetine. In the case of living cells, detergent cannot serve as explanation. It is not unusual that overexpression systems produce partly misfolded protein; thus, part of rSERT may feature non-specific binding sites. In addition, the hypothesized low affinity binding sites may contribute to some degree to the competition resistant signal.

Taken together, these data indicate that all functional features of azidobupramine (**17)** are operative, namely antidepressant-like binding to canonical substrates, UV-induced cross-lining to a known target and furnishing this drug-target complex with a fluorophore as an example for a reporter molecule. Furthermore, it was possible to demonstrate that azidobupramine (**17****)** forms covalent complexes with rSERT not only in rSERT-enriched material but also in living cells stably overexpressing rSERT. Finally, azidobupramine (**17****)** could be competed out of rSERT using paroxetine as competitor.

## Conclusions

To our knowledge, azidobupramine (**17**) is the first non-radioactive structural analogue of tricyclic antidepressants that can be covalently linked to target structures and furnished with reporter molecules while preserving certain antidepressant-like properties. These characteristics are necessary preconditions to conduct extended target identification approaches employing the latest technologies for protein identification. Accordingly, we propose that future studies using azidobupramine (**17**) as model substance have the potential to contribute substantially to a better understanding of the diversity of direct interaction partners of antidepressants. Nevertheless, considerable tasks are ahead, such as the adaptation of the procedures to conditions of endogenous levels of unknown interaction partners. In addition, target identification strategies may benefit from using CuACC to add tags suitable for purification of drug-target complexes, instead of visualization by fluorophores.

## Supporting Information

S1 Fig Uncropped image of the fluorescence analysis of SERT. (PDF)

S2 Fig Uncropped image of the Western analysis of SERT. (PDF)
