# Supplementary material for: Azidobupramine, an Antidepressant-Derived Bifunctional Neurotransmitter Transporter Ligand Allowing Covalent Labeling and Attachment of Fluorophores
Source: PLoS One. 2016 Feb 10;11(2):e0148608. doi: 10.1371/journal.pone.0148608 (PMC4749225; doi:10.1371/journal.pone.0148608)
Supplement: S1 Fig — (PDF) [file pone.0148608.s001.pdf]

S1 Fig. Uncropped image of the fluorescence analysis of SERT.

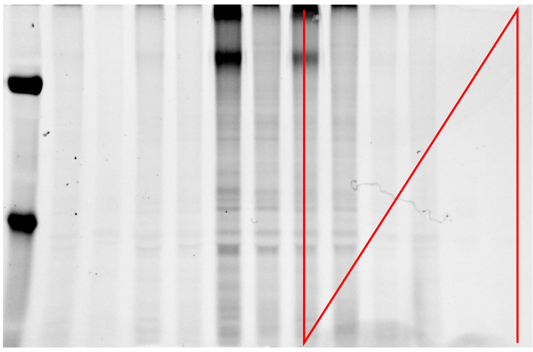

Image Report:

Acquisition Information

Imager	ChemiDoc™ MP
Exposure Time (sec)	30.000 (Auto - Faint Bands)
Flat Field	Applied (Green Epi)
Serial Number	731BR00318
Software Version	4.0.1
Application	Rhodamine
Excitation Source	Green Epi illumination
Emission Filter	605/50 Filter

Image Information

Acquisition Date	08.08.2012 10:51:30
User Name	Werner Anna-Maria
Image Area (mm)	X: 128.4 Y: 83.1
Pixel Size (um)	X: 107.8 Y: 107.8
Data Range (Int)	213 - 28850

Analysis Settings

No analysis performed

S1 Fig. Uncropped image of the fluorescence analysis of SERT. Provided is the original uncropped image from which the bands of the upper panel in the main figure 3 were excised. Included are the parameters describing the image acquisition as documented by the imaging system (ChemiDoc from BioRad). The image contains more lanes than were used for this manuscript, numbered (1-6) are the ones extracted for figure 3.
