# Supplementary material for: Azidobupramine, an Antidepressant-Derived Bifunctional Neurotransmitter Transporter Ligand Allowing Covalent Labeling and Attachment of Fluorophores
Source: PLoS One. 2016 Feb 10;11(2):e0148608. doi: 10.1371/journal.pone.0148608 (PMC4749225; doi:10.1371/journal.pone.0148608)
Supplement: S2 Fig — (PDF) [file pone.0148608.s002.pdf]

S2 Fig. Uncropped image of the Western analysis of SERT.

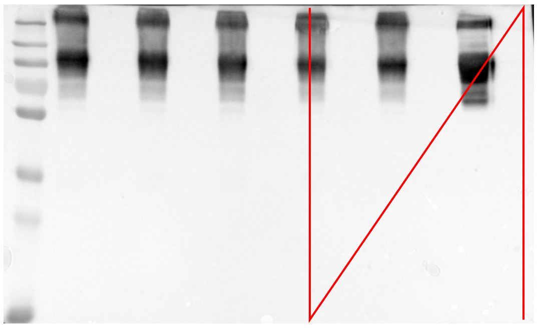

Image Report:

Acquisition Information

Imager	ChemiDoc™ MP
Exposure Time (sec)	4.000 (Manual)
Flat Field	Applied (Lens)
Serial Number	731BR00318
Software Version	4.0.1
Application	Chemi Hi Resolution
Excitation Source	No Illumination
Emission Filter	No Filter
Binning	2x2

Image Information

Acquisition Date	09.08.2012 10:33:39
User Name	annawerner
Image Area (mm)	X: 150.0 Y: 112.1
Pixel Size (um)	X: 215.5 Y: 215.5
Data Range (Int)	0 - 62648

Analysis Settings

No analysis performed

S2 Fig. Uncropped image of the Western analysis of SERT. Provided is the original uncropped image from which the bands of the lower panel in the main figure 3 were excised. Included are the parameters describing the image acquisition as documented by the imaging system (ChemiDoc from BioRad). The image contains more lanes than were used for this manuscript, numbered (1-6) are the ones extracted for figure 3.
